# Adverse events associated with full-thickness resection devices in gastrointestinal endoscopy: National postmarketing surveillance study

**DOI:** 10.1055/a-2820-3777

**Published:** 2026-03-12

**Authors:** Muhammad Shahzil, Talha Bin Kashif, Ali Akram Qureshi, Minahel Shehzadi, Hadie Razjouyan, Ikponmwosa Enofe

**Affiliations:** 1 Internal Medicine, Penn State Health Milton S. Hershey Medical Center, Hershey, United States; 2 8082 The Pennsylvania State University, University Park, United States; 3 66886 Department of Internal Medicine, King Edward Medical University, Lahore, Pakistan; 4 Division of GI-Hepatology and Penn State University Cancer Institute, Penn State Health Milton S. Hershey Medical Center, Hershey, United States; 5 12310 Department of Public Health Sciences, Penn State College of Medicine, Hershey, United States; 6 6429 Department of Gastroenterology and Hepatology, Stanford University, Stanford, United States; 7 Department of Gastroenterology and Hepatology, Penn State Health Milton S. Hershey Medical Center, Hershey, United States

**Keywords:** Endoscopy Upper GI Tract, Endoscopic resection (ESD, EMRc, ...), Precancerous conditions & cancerous lesions (displasia and cancer) stomach

## Abstract

**Background and study aims:**

Endoscopic full-thickness resection (EFTR) using dedicated full-thickness resection devices (FTRDs) offers a minimally invasive option for complex gastrointestinal lesions involving the muscularis propria. Despite growing use, safety data remain limited, which constrains guidelines and uptake.

**Patients and methods:**

We performed a retrospective analysis of FDA MAUDE reports from January 2014 to March 2025. Reports involving FTRDs were reviewed to classify device malfunctions and patient adverse events (AEs). Variables were analyzed using SPSS software.

**Results:**

Sixty-eight FTRD cases were identified. Colonic FTRD was used in 79.4% and gastroduodenal and diagnostic sets in 10.3% each. Device issues (n = 69) included clip non-deployment (79.7%), snare malfunctions (10.1%), and clip detachment (5.8%); grasper malfunctions (2.9%), improper clip placement (1.5%), and thread rupture (1.5%) were also reported. Patient AEs (n = 77) were dominated by colonic perforations (69.5%). Delayed gastric and delayed colonic perforations occurred in three cases each (3.9%). Other events included duodenal perforation (2.6%), hemorrhage (2.6%), and esophageal perforation with mediastinitis (2.6%). Four deaths (5.2%) occurred, two from unrecognized esophageal perforation with mediastinitis and sepsis and two after surgery in patients with significant comorbidities. Surgery was required in 78.7%. Endoscopic clipping alone succeeded in 3.3%. Endoscopic or over the scope clipping followed by surgery was used in 16.4%.

**Conclusions:**

EFTR with FTRD is associated with device malfunctions and patient complications, with colonic perforation being the most frequently reported complication. Careful patient selection and procedural expertise are critical to reduce risk.

## Introduction

Endoscopic procedures have become a cornerstone of gastrointestinal lesion management, providing minimally invasive alternatives for conditions previously considered to require surgery. In 2024, the global endoscopy procedures market was estimated at 191.1 million procedures, with rates projected to increase annually 1 . Endoscopic intervention serves both therapeutic and diagnostic purposes. Lesions identified for endoscopic resection may be epithelial or subepithelial, with specific entities varying by location in the gastrointestinal tract.

Several techniques for resecting gastrointestinal lesions are available to the endoscopist, and selection depends on factors including lesion size, location, depth of invasion, tumor stage, and presence of fibrosis 2 3 4 5 . Endoscopic mucosal resection (EMR) and endoscopic submucosal dissection (ESD) are established methods for resection of lesions confined to the mucosa and superficial submucosa, preserving integrity of the muscularis propria. These techniques, however, face challenges when used for deeper lesions involving the muscularis propria, non-lifting lesions, or lesions with significant fibrosis 3 6 . In this context, endoscopic full-thickness resection (EFTR) has emerged as a minimally invasive procedure that involves concurrent resection and closure of a portion of the gut wall via an endoscopic approach 7 , provided the lesion is of a size that can be accommodated by the device.

EFTR is gaining momentum due to several advantages. Because it enables full-thickness resection, the technique can achieve higher en bloc resection rates, which may confer greater efficacy than EMR and ESD in special cases 8 9 . EFTR can also be used as an adjunct to EMR to achieve even higher en bloc resection rates 10 . The procedure has a high diagnostic yield and facilitates exact risk stratification, which is especially critical for colorectal lesions 11 . In low-risk colorectal lesions, EFTR can obviate need for open surgery, thereby avoiding associated risks and recovery burden 11 12 . The existing literature reports positive results, and EFTR is increasingly being adopted for upper gastrointestinal lesions as well as lower gastrointestinal lesions 13 .

Full-thickness resection devices (FTRDs) are used to perform EFTR. Both EFTR and its devices have undergone considerable evolution 6 9 . Although EFTR can be performed using various endoscopic instruments, including cap-mounted clips, through-the-scope clips, endoscopic plicating devices, endoscopic stapling devices, and needle-catheter knives, the dedicated FTRD (Ovesco Endoscopy, Tuebingen, Germany), introduced in 2014, is cited as the most commonly used apparatus for EFTR 3 14 .

The limited body of literature on EFTR and FTRDs reflects a broader scarcity of data 15 . Consequently, there are no formal guidelines on post-procedure care. Absence of Current Procedural Terminology (CPT) codes for EFTR remains a barrier to adoption across major institutions 3 . This absence may in part stem from limited safety and efficacy data. Because establishment of new procedural codes typically requires evidence of clinical effectiveness, safety, and adoption, real-world data, such as adverse event (AE) reporting from the US Food and Drug Administration (FDA) Manufacturer and User Facility Device Experience (MAUDE) database, and peer-reviewed literature can inform future code development and facilitate broader institutional uptake 16 17 . In short, the paucity of literature surrounding both the technique and the devices presents a barrier to widespread clinical adoption and training 15 . Evidence suggests that robust, data-backed procedures are more readily incorporated into practice, leading to increased adoption, structured training, and institutional acceptance 18 . By contributing real-world safety data through an analysis of AEs reported in the MAUDE database, this study aimed to help fill critical knowledge gaps and support broader implementation of EFTR.

The aim of this study was to conduct a post-marketing formal analysis of real-world reported AEs associated with use of FTRDs for EFTR, utilizing data from the FDA MAUDE database.

## Patients and methods

We analyzed postmarketing surveillance reports on FTRD used to perform EFTR by querying the MAUDE database ( https://www.accessdata.fda.gov/scripts/cdrh/cfdocs/cfmaude/search.cfm ) to identify device-related malfunctions and patient AEs. The MAUDE database compiles reports of suspected device malfunctions, device-associated AEs, patient injuries, and outcomes including death. Reports are submitted by mandatory reporters, comprising manufacturers, importers, and device user facilities, as well as voluntary reporters such as healthcare professionals, patients, and consumers. The database is updated monthly and is used by the FDA and other regulatory agencies to detect safety signals and support postmarketing risk-benefit assessments of medical devices.

For this study, data were queried from January 2014 to March 2025. Data were examined for duplicate reporting and entries with missing or unavailable device problem information were excluded. Suspected duplicate reports were identified by two authors (MS and IE). A third author (AAQ) verified duplicate status in a blinded, unbiased fashion. For entries with improperly coded device problems or adverse outcomes, narrative descriptions were reviewed and data were recoded to maintain consistency in reporting and accuracy in classification.

Statistical analysis was performed using IBM SPSS Statistics 31 (IBM Corp., Armonk, New York, United States, latest available version at the time of analysis). Device-related AEs and patient-related outcomes are presented as frequencies and percentages.

No Institutional Review Board (IRB) approval was required for this study because the data are publicly available and de-identified.

## Results

### Study reports and device use

We identified 68 medical device reports (MDRs) in the FDA MAUDE database involving use of the FTRD that described suspected device-related problems and patient AEs. Individual MDRs could include more than one device problem and more than one patient outcome; therefore, the total number of reported device problems and patient-related AEs exceeds the number of reports.

**Fig. 1:**
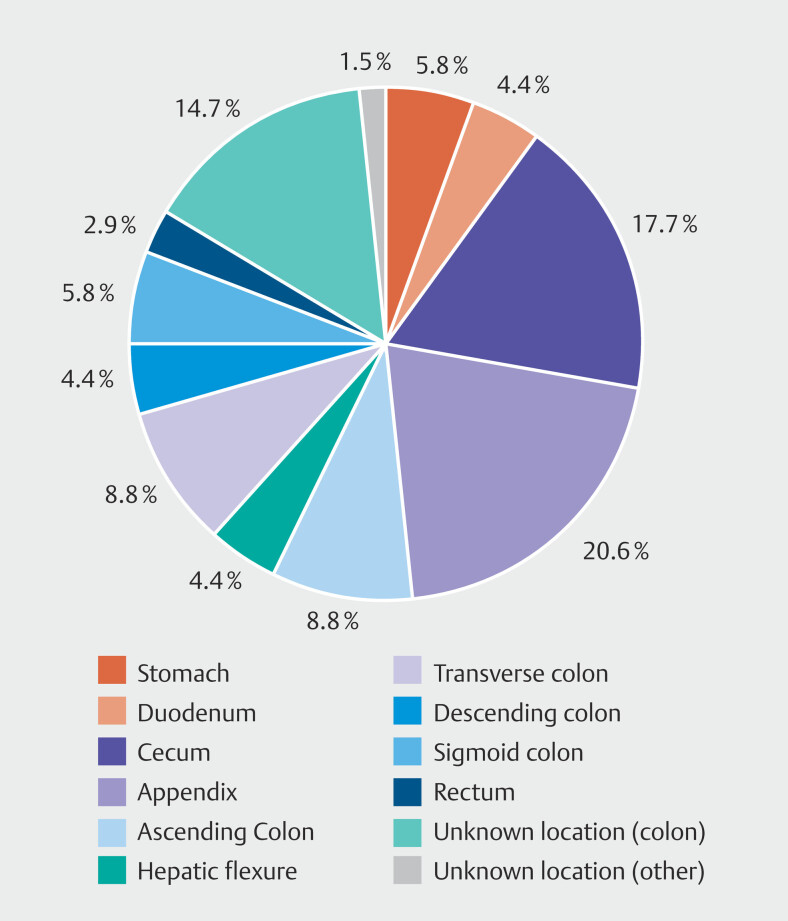
Pie chart showing distribution of FTRD interventions by anatomical location. Most common were appendix (20.6%) and cecum (17.7%), followed by colon not specified (14.7%), stomach (5.8%), duodenum (4.4%), and other colonic segments.

Among the 68 reports, 54 (79.4%) involved the colonic FTRD set, whereas seven reports (10.3% each) involved the gastroduodenal and diagnostic FTRD sets ( Table 1 ). Most reported interventions occurred in the colon (n = 61, 89.7%), with the most frequent locations being the appendix (n = 14, 20.6%) and cecum (n = 12, 17.7%). Upper gastrointestinal interventions were less common, with four reports involving the stomach (5.8%) and three involving the duodenum (4.4%) ( Fig. 1 , Table 2 ).

**Table:** **Table 1** Type of full-thickness resection device used.

	Frequency	Percentage
Diagnostic FTRD set	7	10.3
Gastroduodenal FTRD set	7	10.3
Colonic FTRD set	54	79.4
Total	68	100
FTRD, full-thickness resection device.		

**Table:** **Table 2** Location of full-thickness resection device intervention.

Location	Frequency	Percentage
**Upper gastrointestinal**	**7**	**10.3**
Stomach	4	5.8
Duodenum	3	4.4
Total	7	10.3
**Lower gastrointestinal**	**61**	**89.7**
Cecum	12	17.7
Appendix	14	20.6
Ascending colon	6	8.8
Hepatic flexure	3	4.4
Transverse colon	6	8.8
Descending colon	3	4.4
Sigmoid colon	4	5.8
Rectum	2	2.9
Colon (location not specified)	10	14.7
Unknown location	1	1.5
Total	68	100

### Device-related problems

A total of 69 device-related problems were reported across the 68 MDRs. The most common problem was clip non-deployment, reported in 47 instances (79.7%). In most reports, a definitive mechanical defect was not identified, and the available information did not allow differentiation between device-related, technical, or operator-related factors.

Other reported device-related problems included clip detachment from the resection site (n = 4, 5.8%) and snare-related malfunctions, such as slippage, breakage, or inability to close or resect tissue (n = 7, 10.1%). Additional reported issues included grasper malfunction (n = 2, 2.9%), improper clip placement (n = 1, 1.5%), and thread rupture (n = 1, 1.5%). In seven reports (10.1%), a patient AE was described without an identifiable device-related or use-related problem. All device-related problems are summarized in Table 3 .

**Table:** **Table 3** Problems identified during use of full-thickness resection device.

	Frequency	Percentage
Clip detached from resection site	4	5.8
Grasper caught in clip/tissue/did not deploy properly	2	2.9
Improper clip placement	1	1.5
Clip did not deploy	47	79.7
Thread rupture	1	1.5
Snare/device break	2	2.9
Snare slipped and cut outside of cap	2	2.9
Snare malfunction (could not close/resect tissue/stuck in tissue)	3	4.3
Adverse events without identified device or use problem	7	10.1
Total	69	100
OTS, over-the-scope.		

### Patient-related adverse events

Across the 68 MDRs, 77 patient-related AEs were reported. The majority of AEs involved perforation-related complications, defined as unintended injury or incomplete defect closure resulting in clinical sequelae, such as peritonitis, need for rescue endoscopic therapy, or surgical intervention.

The most frequently reported AE was colonic perforation, occurring in 50 instances (69.5%). Delayed gastric perforation with or without peritonitis and delayed colonic perforation with or without peritonitis were each reported in three instances (3.9%). Additional AEs included duodenal perforation (n = 2, 2.6%), hemorrhage (n = 2, 2.6%), and esophageal perforation with or without mediastinitis (n = 2, 2.6%). In seven reports (9.1%), no patient harm was reported.

**Fig. 2:**
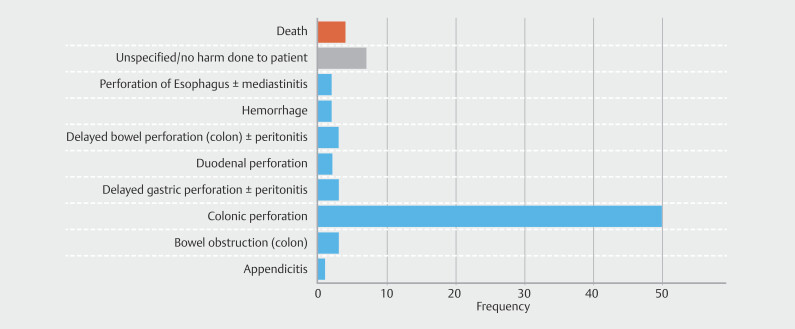
Bar graph of patient complications associated with FTRD use. Colonic perforation dominated (69.5%), with other events including death (5.2%), delayed gastric/colonic perforation (3.9% each), obstruction (3.9%), duodenal perforation (2.6%), hemorrhage (2.6%), and esophageal perforation (2.6%).

Four deaths (5.2%) were described. Two deaths were attributed to mediastinitis and sepsis following unrecognized esophageal perforation. Two additional deaths occurred following surgical intervention in patients with significant comorbidities ( Fig. 2 , Table 4 ).

**Table:** **Table 4** Patient complications resulting from use of full-thickness resection device.

	Frequency	Percentage
Appendicitis	1	1.2
Bowel obstruction (colon)	3	3.9
Colonic perforation	50	69.5
Delayed gastric perforation +/− peritonitis	3	3.9
Duodenal perforation	2	2.6
Delayed bowel perforation (colon) +/− peritonitis	3	3.9
Hemorrhage	2	2.6
Perforation of esophagus +/− mediastinitis	2	2.6
Unspecified/no harm to patient	7	9.1
Death	4	5.2
Total	77	100

### Perforation characteristics

Perforation-related complications were reported in 57 instances across different gastrointestinal locations. Of the 50 colonic perforations, one occurred during device insertion in the sigmoid colon. Delayed gastric perforations were frequently accompanied by peritonitis, accounting for 50% of delayed perforation cases and representing 42.9% of complications reported in the upper gastrointestinal tract.

One report described a patient receiving peritoneal dialysis who developed a delayed colonic perforation 7 days after the procedure; the report noted need to transition from peritoneal dialysis to hemodialysis prior to transmural colorectal interventions. Two esophageal perforations were reported. One occurred during device insertion and the other followed esophageal dilation performed to facilitate device passage in a patient with achalasia. Both cases were complicated by mediastinitis, with one patient requiring surgery and subsequently dying from sepsis.

### Management of adverse events

Among the 61 patients for whom therapeutic management was documented, 48 (78.7%) required surgical intervention. In five cases (8.2%), endoscopic clipping was attempted prior to surgery and in another five cases (8.2%), over-the-scope (OTS) clip placement was performed prior to surgery. Endoscopic clipping alone was successful in two cases (3.3%). In one case (1.6%), the perforation was successfully closed using X TACK endoscopic suturing ( Table 5 ).

**Table:** **Table 5** Therapy for patient complications from use of full-thickness resection device.

	Frequency	Percentage
Endoscopic clipping	2	3.3
Endoscopic clipping and then surgery	5	8.2
OTS clip and then surgery	5	8.2
Surgery	48	78.7
X TACK	1	1.6
Total	61	100
OTS, over-the-scope clip; X TACK, endoscopic suturing device.		

## Discussion

Our analysis included reports citing three types of FTRD sets (diagnostic FTRD set, gastroduodenal FTRD set, and colonic FTRD set), with the colonic FTRD set used in 79.4% of reports. These devices were employed across various locations in the gastrointestinal tract; the most common interventions targeted the appendix (20.6%) and cecum (17.7%). The majority of patient complications were related to perforation after resection. The most common site was the colon (69.5%). Delayed perforations and resultant peritonitis were also reported in the stomach (3.9%) and colon (3.9%). Four deaths (5.2%) were reported after EFTR; one case involved mediastinitis and sepsis due to an unrecognized esophageal perforation, whereas the others were attributed to severe comorbidities following surgery for bowel perforation. The most common technical problems during FTRD use were clip-related: Clip deployment errors (79.7%) were most frequent, followed by clip detachment from the site (2.9%) and improper placement (1.5%). Snare breakage or slipping, thread rupture, and grasper deployment errors were also reported. Most cases with complications were managed with open surgery (78.7%) or with endoscopic clipping or OTS clip placement followed by surgery (16.4%).

Since Schurr and colleagues unveiled the first device for EFTR in 2001 18 for resection of colonic lesions, the potential of EFTR has been well recognized. Indications for EFTR span a wide spectrum from diagnostic resection to lesions suspicious for malignancy, non-lifting adenomas, and lesions infiltrating the muscularis propria 19 . The technique for EFTR centers on two approaches to defect closure (exposed and non-exposed) based on whether the defect is closed after or before resection, respectively 3 . Non-exposed EFTR requires approximation of the serosa from the two margins, creating duplication and then clipping, thereby ensuring closure before resection. In non-exposed EFTR, cap-mounted clip devices such as the OTS clip and Padlock represent advances in defect closure, although their use is restricted to smaller lesions due to cap and clip size.

The combined FTRD (Ovesco Endoscopy) integrates closure and resection into a single platform. An applicator cap carries an OTS clip with increased capacity to accommodate tissue and also houses a snare, tissue grasper, and an insertion balloon to facilitate passage. The grasping device pulls the lesion into the cap, followed by clip deployment and resection with the snare 3 . The FTRD device is effective for both upper and lower gastrointestinal lesions. It was previously approved only for lower gastrointestinal use due to its thickness, which hindered upper gastrointestinal passage 3 . With device developments, including a narrower cap and smaller diameter, multiple studies have reported positive experiences in both locations. In a multicenter study of 56 patients with upper gastrointestinal lesions, Hajifathalian et al. reported technical success in 77% of patients, with R0 margins in 68% 20 . For colonic lesions, Schmidt et al., in a multicenter study of 181 patients, reported a technical success rate of 89.5% and an R0 resection rate of 75% 2 . Similarly, Richter Schrag et al. reported a 75% technical success rate and an 80% R0 resection rate for colonic lesions 21 . Although head-to-head comparisons are lacking, published ESD and submucosal tunnel endoscopic resection (STER) outcomes provide context for interpreting FTRD results. For ESD of gastric subepithelial lesions, perforation rates have been reported as high as 14%. In pooled analyses of STER for upper gastrointestinal subepithelial lesions, R0 resection rates of approximately 94% and perforation rates of approximately 6% have been observed 20 . A recent multicenter cohort study found that EFTR using FTRD after local excision of T1 colorectal cancer with Rx or R1 margins improved disease-free survival compared with completion surgery with lymphadenectomy 22 .

Regarding AEs, our analysis revealed a predominance of perforation-related complications. In contrast, other reports describe substantially lower complication rates. In the upper gastrointestinal tract, Hajifathalian et al. reported 21% mild to moderate AEs, primarily bleeding-related, with no perforations for gastric lesions 20 . Bauder et al. reported a 16% rate of minor bleeding and no perforation or major bleeding events for duodenal lesions 23 . In colonic lesions, Schmidt et al. reported serious AEs in 4.4% of patients 2 , whereas Richter Schrag et al. reported no perforations and one bleeding event in their series 21 . Data from the German and Dutch colorectal FTRD registry encompassing 1,892 procedures demonstrated an overall AE rate of 11.3% and a perforation rate of 2.5%, providing important real-world context for device safety 24 . Because our analysis is not based on individual patient data, it does not reflect incidence; rather, it characterizes types of complications reported within a passive surveillance system. One hypothesis is that perforation may be more likely in colonic than gastric interventions due to differences in epithelial thickness and histology 20 .

Technical problems identified in our analysis were largely clip-related. Prior literature has described challenges with lesion grasping and incorporation into the cap 2 21 , snare failure 22 , loop breakage 23 , and unexpected clip failure 25 . Schmidt et al. reported that snare and cap incorporation issues contributed to difficult resections in 15% of cases 2 . Incomplete resection may occur when the snare is positioned too far from the lesion base, a limitation that has prompted design modifications aimed at improving tissue capture and grip 2 . Clip deployment failure has also been attributed to scope angulation in anatomically challenging or tortuous colons 25 . Clip dislodgement during or after resection may require endoscopic suturing or surgical intervention 18 . In addition, failure of tissue incorporation into the cap can be influenced by lesion size, location, mucosal thickness, access, and scarring from prior interventions 2 . Inherent limitations of our study constrain root-cause attribution. Although clip non-deployment and perforation frequently co-occurred in reported narratives, the available data do not permit reliable distinction between true device malfunction, technical factors, operator-related error, or patient- and lesion-specific contributors. Accordingly, these findings should be interpreted as signal characterization rather than determination of causality. It is also important to note that device refinements have been introduced to improve deployment reliability, including incorporation of a blue ring within the clip holder to visually confirm successful clip release 26 . Because many MAUDE reports lack detailed device versioning and may predate such modifications, reported clip-related events may not reflect current-generation device configuration.

In this context, operator training and experience warrant consideration. Unlike many advanced endoscopic resection tools, use of the FTRD is commonly preceded by structured training and credentialing recommendations, representing a relative strength of this platform in promoting standardized and safe adoption. Given the technical complexity and risk profile of EFTR, multiple expert reviews recommend that these procedures be performed in centers with advanced endoscopy expertise, multidisciplinary support, and formalized training pathways 14 . Proposed EFTR training paradigms include cognitive preparation, simulator-based practice, mentored procedures, and competency assessment prior to independent practice 14 . Such structured approaches may mitigate operator-related technical challenges and improve management of AEs, although these factors cannot be evaluated within the MAUDE dataset. EFTR using the FTRD has been shown to have a relatively short and favorable learning curve compared with other advanced resection techniques. Published clinical series demonstrate that technical success and R0 resection rates improve substantially after approximately 15 to 20 cases, with stabilization thereafter. These observations are supported by both single-center experiences and pooled multicenter analyses 27 28 . Although the MAUDE database does not permit assessment of operator experience, procedure chronology, or volume-outcome relationships, the existing EFTR literature supports presence of a learning curve that may influence technical outcomes and complication profiles during early adoption 27 28 .

There is a lack of direct comparative studies between FTRD-based EFTR and techniques such as ESD, EMR, or surgical resection. Although this represents an avenue for future research, available evidence suggests that EFTR using FTRD can yield favorable outcomes with reduced morbidity in appropriately selected patients when performed by trained operators.

We acknowledge several important limitations. First, this analysis is based on reports from the FDA MAUDE database, for which reporting is non-standardized and subject to variable data quality. Because MAUDE is a passive surveillance system populated through both mandatory and voluntary reporting, it is inherently subject to under-reporting, preferential reporting of severe or unexpected outcomes, incomplete narratives, and variable verification of reported events. Presence of a report does not establish causality, and devices are not consistently available for independent evaluation. In addition, follow-up submissions may overwrite earlier reports, and certain data elements may be redacted under Freedom of Information Act exemptions, limiting accurate reconstruction of event chronology. Second, MAUDE data are not intended to estimate AE incidence, evaluate temporal trends, or compare event rates across devices. Reports frequently lack complete procedure details, patient characteristics, and operator level information, including experience or training background. Although we attempted to explore temporal patterns, absence of denominator data and precise procedure chronology precluded meaningful assessment of changes over time. Therefore, we were unable to evaluate differences in serious AEs between early and later procedure periods. Finally, although a high number of perforations and device malfunctions were reported, no definitive correlation could be established, demonstrating inherent limitations of MAUDE data in distinguishing device-related factors from operator or patient-related contributors.

## Conclusions

This analysis of FDA MAUDE reports characterizes AEs and technical issues reported with use of FTRDs in real-world practice. Reported complications were predominantly perforation and clip-related technical problems, requiring surgical management. These findings do not represent incidence rates and should be interpreted as safety signals within a passive surveillance system. When considered alongside prospective studies and large registry data demonstrating low overall AE rates, the results highlight the importance of appropriate patient selection, structured training, and procedure expertise. Continued device refinement and complementary data from prospective registries will be essential to further optimize safe use of FTRD-based EFTR.
